# Bioactive Compounds from Leaves and Twigs of Guayule Grown in a Mediterranean Environment

**DOI:** 10.3390/plants9040442

**Published:** 2020-04-02

**Authors:** Giovanna Piluzza, Giuseppe Campesi, Maria Giovanna Molinu, Giovanni Antonio Re, Leonardo Sulas

**Affiliations:** 1 National Research Council, Institute for the Animal Production System in Mediterranean Environment, Traversa La Crucca 3, località Baldinca, 07100 Sassari, Italy; giovanna.piluzza@cnr.it (G.P.); giuseppecampesi2003@gmail.com (G.C.); giovanniantonio.re@cnr.it (G.A.R.); 2 National Research Council, Institute of Sciences of Food Production, Traversa La Crucca 3, località Baldinca, 07100 Sassari, Italy; mariagiovanna.molinu@cnr.it

**Keywords:** *Parthenium argentatum*, non-rubber byproduct, phenolic compounds, industrial crop

## Abstract

Guayule (*Parthenium argentatum*) is one of the most promising alternatives to produce natural rubber. As the guayule leaves represent a waste byproduct in the rubber extraction process, their exploitation might contribute to the valorization of the whole plant biomass. The specific aim of our study was to determine the antioxidant capacity and the content of phenolic compounds in leaves and twigs of different guayule lines cultivated in a Mediterranean environment. The antioxidant capacity and the contents of phenolic were affected significantly by guayule accession and harvest time. Overall means in twigs represented about 60% of the corresponding leaf values for antioxidant capacity as DPPH (1,1-diphenyl-2-picrylhydrazyl) and not tannic phenolic and about 55% as ABTS (2,2′-azinobis (3-ethylbenzothiazoline-6-sulphonic acid) diammonium salt), total phenolic, tannic phenolic and total flavonoid contents. Five individual phenolic compounds were identified in methanolic extracts of leaves. Neochlorogenic acid (62.5–174.8 mg g^−1^ DW) and chlorogenic acid (28.6–41.1 mg g^−1^ DW) were the most abundant phenolic acids. In addition to the compounds quantified in leaves, cynarin was identified only in twigs and for the first time in guayule biomass. Our results indicate that guayule leaves and twigs represent a rich source of antioxidants for potential applications in fodder, nutraceutical and pharmacological sectors.

## 1. Introduction

Natural rubber, for its excellent and unique properties, represents a strategic raw material whose demand is increasing worldwide [1]. Traditionally, it is extracted as latex from the plantations of *Hevea brasiliensis* (Willd. ex A. Juss.) Müll. Arg, which are mainly grown in tropical areas of Asian–Pacific countries and supply 90% of the global production [2]. 

However, potential shortages in natural rubber, caused by several factors (fungi attacks, land use change in production areas, vulnerability to climate change, etc.) and the onset of allergies due the presence of some proteins in its latex affect availability and use of Hevea rubber [3]. Since 1997, European Union has listed natural rubber as a critical material and imports 1.2 million tons each year [4]. 

These shortcomings and the rising global demand in natural rubber have cyclically re-addressed interest in alternative sources, of which the most important are represented by guayule (*Parthenium argentatum* A.Gray) and Kazakh dandelion (*Taraxacum kok-sagyz* L.E.Rodin) [3]. 

Guayule is a shrub from the Asteraceae family native to Northern Mexico and Southwest regions of the USA. As it grows within a range of temperature from −15 to +40 °C and an annual rainfall requirement of 350 to 800 mm [5], guayule is well suited to semi-arid and Mediterranean regions.

Currently, guayule is considered as an alternative source, having the potential to become commercial crop, to produce natural rubber in Europe. Sfeir et al. [6] evaluated technical and economic feasibility of a guayule commodity chain in Mediterranean Europe, indicating that this crop might be a sector commercially profitable only in the framework of a biorefinery approach. This requires the valorization of the whole plant biomass and that of the production of bioenergy and non-rubber co-products, to be converted into products and/or raw materials with high added value for different sectors [7]. Therefore, it is justified an overall interest in developing new agricultural commodities such as guayule for Mediterranean or semi-arid climates over the next years [8]. 

As guayule leaves contain negligible amounts of rubber compared to branches, they represent a waste by-product [9] in the rubber extraction processes from its biomass. In fact, the complete removal of leaves before homogenization is a necessary step to produce high-quality latex, avoiding fine solids contamination mostly attributable to the co-homogenized leaves [10]. As regards the presence of bioactive compounds in leaves of guayule, Mears [11] reported the distribution of 34 flavonoids detected in leaves of North American *Parthenium* spp. A recent paper focused on procedures for the extraction of bioactive compounds from leaves of guayule [12]. 

Despite the abundance of literature dealing with studies on guayule for rubber production and the shared approach for the exploitation of all biomass fractions, very little information is available regarding secondary plant metabolites and, in particular, the phenolic fraction contained in its leaves. Concurrently, no information dealing with the phenolic fraction in twigs (i.e., young thin stems) of guayule, as well as the possible influence of genotype and harvest time seems obtainable.

Plant bioactive compounds have been widely studied as natural phytochemicals due to superior antioxidant properties compared with synthetic antioxidants. Indeed, bioactive compounds from leaf extracts of tree species have been investigated for the beneficial effects (antioxidant, anticancer, anti-inflammatory, antidiabetic, etc.) on human health [13]. Therefore, the investigation, identification and quantification of the bioactive compounds of leaf extracts are highly important for its antioxidant properties and related health effects, of which phenolic compounds are primarily responsible for [14]. Additionally, phenolic compounds play a role in plant defense reactions against biotic and abiotic stresses, as well as in animal nutrition and welfare. [15,16]. 

Within a research framework started in Sardinia (Italy) to evaluate adaptation and performances of guayule lines cultivated under Mediterranean conditions, our study focused on the exploitation of guayule leaves and twigs, as source of phenolic compounds and antioxidants for potential applications in fodder, nutraceutical and pharmacological sectors. Therefore, the specific aims of our study were to determine, in the above-mentioned plant fractions, the antioxidant capacity and content of phenolic compounds as well as their variations due to different genotypes and harvest times.

## 2. Results and Discussion

### 2.1. Antioxidant Capacity and Phenolic Content

Phenolic compounds are secondary plant metabolites, which have been classified into several classes, namely, flavonoids, phenolic acids, stilbenes and lignans, according to their chemical structure. They are of pivotal importance in regulating plant defenses mechanisms against several stresses and in healing wounds of rubber plants. Additionally, many studies have explored the pharmacological benefits of phenolic compounds, including the high antioxidant potential and other health-promoting properties [14]. In leaves, the antioxidant capacity and the contents of total phenolics (TotP), non-tannic phenolics (NTP) and total flavonoids (TotF) were affected significantly by both guayule accession and harvest time (Table 1). Line P803 showed the highest antioxidant capacity values in winter. The content of TotP ranged from 147 to about 204 g gallic acid equivalent (GAE) kg^−1^ in P803, the only accession where the effect of harvest time was significant. The same trend was observed for NTP values.

Overall average for tannic phenolics (TP) content was unaffected by line and season. AZ-1, P803 and 11591 exhibited the highest TotF values in winter and spring, whereas a marked seasonal increase was noticed for AZ-2. 

In twigs, the harvest time did not affect ABTS (2,2′-azinobis (3-ethylbenzothiazoline-6-sulphonic acid) diammonium salt) and NTP values, whereas TP values were irrespective of guayule line (Table 2). At both harvest times, the highest values for the antioxidant capacity and the contents of TotP were found in AZ-1 and AZ-2. The peak values of NTP were reached in AZ-2 and AZ-1, in winter and spring, respectively. A remarkable seasonal increase in TotF content was recorded for P803. Overall means in twigs represented about 60% of the corresponding leaf values for antioxidant capacity (DPPH) and NTP and about 55% for ABTS, TotP, TP and TotF contents (Table 1 and Table 2).

For guayule leaves, significant correlations were found between the antioxidant capacity by means of ABTS and DPPH (1,1-diphenyl-2-picrylhydrazyl) methods and the phenolic contents (Table 3). DPPH and total phenolics showed a highly significant correlation (R^2^, 0.6774). Significant correlations were also established between antioxidant capacity and NTP contents. Regarding twigs, closer correlations were found than in leaves. In fact, significant correlations were found between the antioxidant capacity by means of the ABTS and DPPH methods and the phenolic content reaching a significant correlation of R^2^, 0.8581 and 0.9174, respectively (Table 3).

Our results agree with other studies that reported the relationship between antioxidant capacity and total phenolic compounds in species belonging to the Asteraceae family such as Kazakh dandelion and *Taraxacum officinale* Weber ex Wiggers, as well as in many representative herbs within Labiatae and Asteraceae [17,18,19,20]. Unfortunately, very few papers deal with chemical composition and antioxidant capacity in leaves of guayule. Banigan et al. [21] investigated crude protein content of deresinated and derubberized leaf tissue and the amino acid composition. Mouse feeding studies showed deresinated leaf meal to be nontoxic but deficient as a sole source protein. Leaf bromatological composition of guayule is being investigated by Sulas et al. [22]. Mears [11] determined the distribution of flavonoids detected in the North American species belonging to *Parthenium* genus. *P. argentatum* showed a flavonoid pattern rich in aglycones and glycosides: derivatives of quercetagetin, 6-hydroxykaempferol, quercetin, kaempferol and kaempferol 3-methyl ether. 

It is noteworthy that our values for total phenolics reported in Table 1 were up to 7-fold or even 11-fold higher than leaf peak values indicated by Piana et al. [12]. According to that paper, dry guayule leaf material from Mexico was tested for both solid-liquid and ultrasounds-assisted extractions according to a range of conditions for green solvent composition and extraction time. Even if the total phenolic content of extracts was quantified by the Folin–Ciocalteu’s assay as in our study, the magnitude of differences might be, presumably, explained by differences in the tested genotype, growth environment, sampling and storage procedures. Conversely, our samples were collected on site and immediately frozen in liquid nitrogen, then stored at −80 °C, and afterwards freeze-dried. An important implication arising from this direct comparison is that the potential of guayule leaves as source of total phenolic and antioxidants, based on our results, is markedly higher than that reported so far. For flavonoids quantification, a different method and standard were used, so a quantitative comparison of data is difficult. Regarding comparison with other species belonging to same genus, Panwar et al. [23] found in the weed *Parthenium hysterophorus* L. a content of phenolics of 20.8 mg GAE g^−1^, but they performed the quantification with an alkaline hydrolysis method. Within the same botanical family, Molinu et al. [20] found in *Taraxacum kok-saghys* leaves a content of antioxidant capacity, total phenolics, total flavonoids of 19.6 mmol Trolox equivalent antioxidant capacity (TEAC) 100 g^−1^ DW, 106.4 g GAE kg^−1^ DW, 22.9 g catechin equivalent (CE) kg^−1^ DW respectively, lower than our results. A study dealing with bioactive compounds and antioxidant capacity from Mediterranean garland (*Chrysanthemum coronarium* L.) showed in leaves a content of antioxidant capacity (14.4 mmol TEAC 100 g^−1^) lower than our results, and total flavonoids (32 g CE kg^−1^ DW) very close to our results [24]. Sardinian milk thistle (*Silybum marianum* (L.) Gaertn.) populations were studied for chemical and productive properties by Sulas et al. [19] reporting, in acetone/water leaves extract, a content of antioxidant capacity (7.6 mmol TEAC 100 g^−1^ DW) and total flavonoids (11.5 g CE kg^−1^ DW) lower than our results. A content of phenolic compounds lower (18.7 mg GAE g^−1^ DW) than our results was also reported by Benchaachoua et al. [25] in a aqueous-methanol extract of leaves of *S. marianum* collected in Algeria. Ben Salem et al. [26] found phenolic contents of artichoke (*Cynara scolymus* L.) leaves extracts from 39.9 to 54.5 mg GAE g^−1^ DW lower than our results. Outside the Asteraceae family, a comparison with the legume shrub tagasaste (*Chamaecytisus proliferus* var. *palmensis*) grown in the same site than guayule, revealed that, at late autumn cutting, the antioxidant capacity and total flavonoids were quite similar to guayule results for leaves, but different for twigs [27]. Overall, comparisons with other study regarding the similar species are very difficult due to variations in methods, procedures and standard used for the analysis quantification According to the literature, seasonal changes in the phenolic content of plant leaves might depends on species, site conditions (altitude, solar radiation), the position of the leaves and variety, etc. [28,29,30]. 

### 2.2. RP-HPLC Analysis of Phenolic Compounds

Twenty-eight individual phenolic compounds were screened by RP-HPLC based on the literature concerning phenolic composition in *Parthenium* genus and other members of the Asteraceae family. Five individual phenolic compounds were identified in leaf methanolic extracts of guayule. Identification of compounds was carried out by comparison with their corresponding standard and with data reported by other authors. The five individual compounds identified in leaves were neochlorogenic (5-caffeoylquinic acid), chlorogenic (3-caffeoylquinic acid), cryptochlorogenic (4-caffeoylquinic acid), isovanillic, and 3,5-DCQ (3,5-Di-O-caffeoylquinic acid) acids (Table 4). Statistical analysis evidenced variations due to accession and harvest time. The neochlorogenic (62.5–174.8 mg g^−1^ DW) and chlorogenic acids (28.6–41.1 mg g^−1^ DW) were the most abundant phenolic acids, which taken together contributed on average to about 92% of the quantified total cinnamic acid derivatives. The neochlorogenic, cryptochlorogenic and 3,5-DCQ acids were significantly affected by harvest time in AZ-1, P803 and AZ-2, respectively, whereas chlorogenic and isovanillic acids were irrespective of harvest time. In particular, the spring content of neochlorogenic acid only represented 38% of the corresponding winter value in P803, whereas the spring content of 3,5-DCQ showed a 72% increase compared to the winter value in AZ-2. In guayule leaves, Piana et al. [12] identified chlorogenic and isovanillic acids as our results, and others caffeoylquinic acid isomers, identified in our study as criptochlorogenic and neochlorogenic acids. In addition, the same authors detected different dicaffeoylquinic acid isomers, identified as 3,5-DCQ and cynarin (1,3-Di-caffeoylquinic acid) in our study. Chlorogenic, caffeic and vanillic acids were also detected in leaves and flowers of *P. hysterophorus* grown in different regions of Ethiopia [31]. On the contrary, caffeic acid was not detected in our study. Moreover, a review of Khan and Ahnmad [32] reported the presence of chlorogenic, neochlorogenic, vanillic acids in leaves of *P. hysterophorus*. Chlorogenic acid (lower than 0.2 μg g^−1^) was also detected in the ethanolic extract of aerial parts of *Chrysanthemum parthenium* Bernh, very low value, compared to our results, probably due to a different solvent extraction [33]. A study regarding *Bellis perennis* L. revealed that a greater amount of chlorogenic and neochlorogenic acids were detected in leaves than in root; these compounds were studied for their allelopathic properties [34]. 

Derivatives of hydroxycinnamic acid, and particularly chlorogenic acid, are considered a characteristic trait of the Asteraceae plants, although they are ubiquitous of the plant kingdom [34,35]. Moreover, chlorogenic acid is an important and biologically active dietary polyphenol, playing several important and therapeutic roles in human health, such as antioxidant, antibacterial, hepatoprotective, cardioprotective, anti-inflammatory, antipyretic, neuroprotective, anti-obesity, antiviral, anti-microbial, anti-hypertension, free radical scavenging and central nervous system stimulator activity [36]. The same authors referred that chlorogenic acid has shown hepato-protective effects in animals; its positive effect was also revealed on performance and antioxidant function in piglets [37]. Cynarin was identified only in guayule twigs, in addition to the same five individual compounds quantified in leaves (Table 5), and for the first time in guayule biomass. Again, statistical analysis evidenced variations due to accession and harvest time. Overall, a dramatic decrease in neochlorogenic acid content in twigs compared to leaves was detected. Taken together chlorogenic and 3,5-DCQ acids contributed to about 95% of the total cinnamic acid derivatives that were quantified in guayule twigs. Spring reduction were recorded in all accessions for neochlorogenic acid, in AZ-2 and 11591 for chlorogenic acid, in AZ-1 and 11591 for isovanillic acid and in 11591 for 3,5-DCQ acid, respectively.

Conversely, within the Asteraceae family, cynarin was detected in methanolic extract of artichoke leaves as reported by Farhan et al. [38]. This compound was also identified in methanolic extract of sunflower (*Helianthus annuus* L.) sprouts, showing antioxidant and antidiabetic effects in vitro models [39]. It is noteworthy that cynarin has even been reported as anti-HIV active [40].

In leaves, the antioxidant capacity with both ABTS and DPPH assays was positively correlated with neochlorogenic and isovanillic acids, whereas chlorogenic acid was correlated with Ptot and NTP (Table 6). The relatively higher concentration of neochlorogenic acid was the most probable determinant for observed antioxidant activities in leaves. In twigs, all the six identified compounds were weakly correlated with ABTS and four of them with DPPH. Only chlorogenic and 3,5-DCQ acids were also correlated with TotP, NTP, TP and TotF. According to Kozyra et al. [41], chlorogenic acid significantly influence the antioxidant activity of methanolic extracts from inflorescences of *Carduus* sp. In Loquat (*Eriobotrya japonica* Thunb. Lindl.) fruits, contents of chlorogenic and neochlorogenic acids were significantly correlated with antioxidant capacity, assessed by DPPH and ABTS radical scavenging activity [42].

## 3. Materials and Methods

The field experiments were carried out in Southern Sardinia (39°31′ N, 8°51′ E, Italy), where the climate is Mediterranean with mild winter. The area has a long-term average annual rainfall of 446 mm received mainly in autumn and winter months, and a mean annual air temperature of 17.6 °C. The soil, classified as Typic Fluvaquents, is sandy-clay-loam, with pH 7.8, average nitrogen content of 1.1‰ and phosphorous 16.2 ppm.

Four guayule accessions kindly provided by the Agricultural Research Service of the United States Department of Agriculture (ARS-USDA) were evaluated: the improved guayule lines AZ-1 and AZ-2, the accession P803, and the line 11591, which was developed during the Emergency Rubber Project and used as control. Additional details on the above-mentioned plant materials are reported by Ilut et al. [43]. Seeds were treated to break dormancy using the method of Naqvi and Hanson [44] and were sown in wet filter paper inside a growth chamber. Two weeks after germination, seedlings were moved into jiffy pots and afterwards put in plastic bags. Plantlets were then moved outdoor and transplanted in the field in May 2015. The experimental design was a randomized complete block with three replicates. Seedlings were transplanted in row spaced at 1 m and 0.5 m within the row. Each plot consisted of eight rows (two for each line). Additional outer rows were also transplanted as border. Seedlings were irrigated daily during the first week after transplanting and then at ten-day intervals in summer months from June to early October by using a drip irrigation system. Fertilizer was applied only at the time of field preparation when 30 kg N ha^−1^ was incorporated into the soil.

### 3.1. Leaves and Twigs Sampling

At 30 and 33 months after transplanting, corresponding to winter 2017 and spring 2018, respectively, three plants of each line per plot were cut at ground level and harvested. Aboveground plant dry matter was partitioned into leaves, twigs (i.e., the youngest apical stem portion, 6 to 8 mm in diameter, developed in last growing season), and remaining branches. Leaf and twigs samples were immediately frozen in liquid nitrogen, and stored at −80 °C, until lyophilization with Heto Lyolab 3000 (Heto-Holten A/S, Allerød, Denmark) for 48 h (−55 °C). Leaf and twig subsamples were ground in a mill to a fine powder and stored at −20 °C until analysis. Sample preparation procedures were performed according to Molinu et al. [20]. All samples were analyzed in triplicate.

### 3.2. Phenolic Content and Antioxidant Capacity

Total phenolics (TotP), non-tannic phenolics (NTP) and tannic phenolics (TP) of extracts were determined using the Folin–Ciocalteau reagent, according to procedures previously described [30]. Results were expressed as g of gallic acid equivalent (GAE) kg^−1^ dry matter of plant material (g GAE kg^−1^ DM) by means of a calibration curve of gallic acid. Total flavonoids (TotF) were quantified by colorimetric assay with the AlCl_3_ method, following procedures previously reported [30]. TotF were quantified by catechin calibration curve and results were expressed as g of catechin equivalent (CE) kg^−1^ dry matter (g CE kg^−1^ DM). Antioxidant capacity was evaluated by ABTS (2,2′-azinobis (3-ethylbenzothiazoline-6-sulphonic acid) diammonium salt) and by DPPH (1,1-diphenyl-2-picrylhydrazyl) assays [30]. Trolox (6-hydroxy-2,5,7,8-tetramethylchroman-2-carboxylic acid) was used as the reference standard. For each assay, 0.1 mL of appropriately diluted methanolic extracts was used and a calibration standard curve with Trolox (2-12 μM; R^2^ = 0.997 for DPPH assay and R^2^ = 0.998 for ABTS assay) was made. Briefly, for ABTS assay, 3.9 mL of the ABTS radical solution were mixed with the sample. The spectrophotometric readings were carried out after 6 min at 734 nm. For DPPH assay, sample was mixed with 3.9 mL of DPPH 60 µM and stored in the dark for 120 min, and the absorbance was recorded at 515 nm. The results were expressed in terms of Trolox equivalent antioxidant capacity (TEAC), as mmol Trolox equivalents 100 g^−1^ dry weight of leaves (mmol TEAC 100 g^−1^ DW).

### 3.3. RP-HPLC Analysis of Phenolic Compounds

RP-HPLC method was used to determinate phenolic compounds by an Agilent 1260 series HPLC instrument (Agilent Technologies, Palo Alto, CA, USA) equipped with a quaternary pump (G1311B), degasser, column thermostat (G1316A), auto-sampler (G1329B) and diode array detector (G1315 B, DAD). Chromatographic separation was carried out according to Molinu et al. [20]. The column was a Zorbax Eclipse plus C18 (250 × 4.6 mm, 5 µm; Agilent). Column temperature was set to 30 °C and the flow rate was 0.8 mL min^−1^. The injection volume was 10 μL and the detection wavelengths were set to 280, 330 and 350 nm. Data were processed using the Agilent OpenLAB CDS ChemStation edition 2012. Peak assignment of phenolic compounds was carried out comparing their retention times and UV-vis spectra with those of analytically pure standard (Table 7), and as well as by adding the standard solution to the sample.

### 3.4. Data Analyses

Laboratory measurement values (http://dx.doi.org/10.17632/9zsgwd4gp6.1) were subjected to a two-way analysis of variance, using Statgraphics Centurion XVI version [55] to test the effects of guayule accession, harvest time and their interaction on the following variables: concentrations for antioxidant capacity, total phenolics, total flavonoids and individual phenolic compounds. Differences between means were assessed with the Fisher’s least significant difference (LSD) procedure for means separation. The significance level was fixed at *p* ≤ 0.05 for all the statistical analyses. The regression analyses between polyphenols and antioxidant capacity were calculated using Microsoft Excel 2016.

## 4. Conclusions

The current study is the first report regarding phenolic content, antioxidant activity, and quantification of individual phenolic compounds from biomass fractions of guayule plants grown in a Mediterranean environment.

Our research evidenced that the antioxidant capacity and the content of total bioactive compounds in leaves and twigs were affected significantly by guayule accession and harvest time. However, the improved lines AZ-1 and AZ-2 showed higher contents of total phenolic in twigs, irrespective of harvest time. The cinnamic acid derivatives namely neochlorogenic and chlorogenic acids in leaves and chlorogenic and 3,5-Di-caffeoylquinic acids in twigs were the main antioxidants isolated from guayule extracts.

Therefore, leaves and twigs, the latter at a lower extent, represent a rich source of bioactive compounds in guayule for potential exploitation in fodder, nutraceuticals and pharmacological sectors. Additional studies are needed to characterize biological activities of these extracts.

## Figures and Tables

**Table 1:** Trolox equivalent antioxidant capacity (TEAC) by 2,2′-azinobis (3-ethylbenzothiazoline-6-sulphonic acid) diammonium salt (ABTS) and 1,1-diphenyl-2-picrylhydrazyl (DPPH) methods, total phenolics (TotP), non-tannic phenolics (NTP), tannic phenolics (TP), total flavonoids (TotF) in leaves of the examined accessions of *Parthenium argentatum* harvested at 30 (winter) or 33 months (spring) after transplanting.

	TEAC (mmol 100 g^−1^ DW)						TotP (g GAE kg^−1^ DW)			NTP (g GAE kg^−1^ DW)			TP (g GAE kg^−1^ DW)			TotF (g CE kg^−1^ DW)		
	ABTS			DPPH														
	Winter	Spring		Winter	Spring		Winter	Spring		Winter	Spring		Winter	Spring		Winter	Spring	
AZ-1	23.2 ± 1	20.2 ± 1a	ns	27.4 ± 0.4b	21.3 ± 0.6a	*	181.9 ± 2a	167.4 ± 7b	ns	129.7 ± 4	108.2 ± 1b	ns	52.2 ± 7	59.2 ± 8	ns	39.5 ± 2b	40.7 ± 1b	ns
AZ-2	23.1 ± 0.9	20.8 ± 1a	ns	23.3 ± 2a	25.6 ± 2b	ns	172.3 ± 7a	186.6 ± 8b	ns	113.4 ± 6	115.6 ± 5b	ns	58.9 ± 4	71.0 ± 13	ns	25.9 ± 2a	43.8 ± 1b	*
P803	25.1 ± 1	17.2 ± 2a	*	29.3 ± 0.8b	20.8 ± 1.5a	*	203.7 ± 7b	147.1 ± 6a	*	124.1 ± 6	84.0 ± 8a	*	79.5 ± 10	63.1 ± 5	ns	39.2 ± 3b	31.5 ± 4a	*
11591	26.7 ± 0.4	23.5 ± 0.8b	ns	27 ± 0.6b	27.8 ± 0.4b	ns	183.4 ± 2a	198.7 ± 3b	ns	128.8 ± 8	130.9 ± 8b	ns	58.4 ± 14	67.6 ± 6	ns	38.8 ± 2b	41.8 ± 2b	ns
Avg.	22.5			25.3			180.1			116.8			63.7			37.6		
G × H	ns			**			**			*			ns			**		

In the columns of each harvest (H) time (winter and spring), means followed by the same letter are not significantly different at *p* ≤ 0.05. In the rows, least significant differences test indicates harvest time effect on each examined accession of *Parthenium argentatum*. G × H: interaction between genotype and season of harvest. ns, not significant; ** *p* ≤ 0.001, * *p* ≤ 0.05.

**Table 2:** Trolox equivalent antioxidant capacity (TEAC) by ABTS and DPPH methods, total phenolics (TotP), non-tannic phenolics (NTP), tannic phenolics (TP), total flavonoids (TotF) in twigs of the examined accessions of *Parthenium argentatum* harvested at 30 (winter) or 33 months (spring) after transplanting.

	TEAC (mmol 100 g^−1^ DW)						TotP (g GAE kg^−1^ DW)			NTP (g GAE kg^−1^ DW)			TP (g GAE kg^−1^ DW)			TotF (g CE kg^−1^ DW)		
	ABTS			DPPH														
	Winter	Spring			Winter	Spring	Winter	Spring		Winter	Spring		Winter	Spring		Winter	Spring	
AZ-1	12.4 ± 0.5b	14.1 ± 0.5b	ns	15.9 ± 0.3b	19.9 ± 0.4c	*	102.4 ± 2b	127.7 ± 3b	*	73.9 ± 3a	83.8 ± 8b	ns	28.4 ± 5	43.9 ± 8	ns	22.4 ± 0.5b	28.7 ± 3.5b	ns
AZ-2	14.9 ± 0.3c	14.7 ± 11b	ns	18.3 ± 0.5c	18.8 ± 1.3c	ns	116.8 ± 3b	128.8 ± 7b	ns	85.8 ± 4b	74.9 ± 12a	ns	31 ± 3	53.9 ± 5	*	24.5 ± 1b	22.4 ± 2a	*
P803	10.1 ± 0.8a	11.5 ± 0.8a	ns	11.8 ± 0.6a	14.9 ± 0.4b	*	81.1 ± 6.2a	101.6 ± 6a	*	57.8 ± 10a	59.7 ± 2.1a	ns	23.2 ± 6	41.9 ± 5	ns	12.3 ± 0.2a	20.8 ± 1.3a	*
11591	12.2 ± 0.1b	10.5 ± 0.6a	ns	15.5 ± 0.5b	13.6 ± 0.2a	*	103.4 ± 4b	87.6 ± 6a	*	69.4 ± 4a	55.3 ± 6a	ns	33.6 ± 8	32.3 ± 5	ns	20.3 ± 1b	20.9 ± 2a	ns
Avg.	12.5			16.1			106.1			70.1			36.0			21.5		
G × H	*			***			**			ns			ns			**		

In the columns of each harvest (H) time (winter and spring), means followed by the same letter are not significantly different at *p* ≤ 0.05. In the rows, least significant differences test indicates harvest time effect on each examined accession of *Parthenium argentatum*. G × H: interaction between genotype and season of harvest. ns, not significant; *** *p* ≤ 0.0001, ** *p* ≤ 0.001, * *p* ≤ 0.05.

**Table 3:** Correlations (R^2^) established between total phenolics (TotP), non-tannic phenolics (NTP), tannic phenolics (TP), total flavonoids (TotF) and antioxidant capacity (ABTS, DPPH) in leaves and twigs of the examined accessions of *Parthenium argentatum* harvested at 30 (winter) and 33 months (spring) after transplanting.

	Leaves			Twigs		
	ABTS	DPPH	TotP	ABTS	DPPH	TotP
DPPH	0.4791 **			0.8415 ***		
TotP	0.4643 **	0.6774 ***		0.8581 ***	0.9174 ***	
NTP	0.5918 ***	0.5660 ***	0.6266 ***	0.7620 ***	0.6877 ***	0.6136 ***
TP	0.0034 ns	0.0670 ns	0.2198 *	0.2092 *	0.3128 *	0.4639 **
TotF	0.0151 ns	0.1897 *	0.2444 *	0.4425 **	0.6946 ***	0.5649 ***

ns = not significant; *** *p* ≤ 0.0001, ** *p* ≤ 0.001,* *p* ≤ 0.05.

**Table 4:** HPLC analysis of phenolic acids (mg g^−1^ DM) in leaves of the examined accessions of *Parthenium argentatum* harvested at 30 (winter) or 33 months (spring) after transplanting. (Means ± SD, *n* = 3, Fisher’s test).

	Neochlorogenic Acid			Chlorogenic Acid			Cryptochlorogenic Acid			Isovanillic Acid			3,5-DCQ ^[1]^		
Retention Time (min)	9.46			11.27			11.57			14.1			24.4		
	Winter	Spring		Winter	Spring		Winter	Spring		Winter	Spring		Winter	Spring	
AZ-1	174.8 ± 7.9b	124.9 ± 3.8c	*	33.7 ± 3.5	38.6 ± 3.3b	ns	0.8 ± 0.07	0.93 ± 0.2b	ns	0.5 ± 0.05b	0.4 ± 0.1b	ns	13.9 ± 1.6b	12.2 ± 0.2a	ns
AZ-2	127.9 ± 5.6a	135.8 ± 10.2c	ns	31.6 ± 4.1	41.1 ± 2.2b	ns	0.9 ± 0.05	0.95 ± 0.04b	ns	0.7 ± 0.04b	0.51 ± 0.04b	ns	8.3 ± 1.3a	14.3 ± 0.7ab	*
P803	164.5 ± 11.7b	62.5 ± 0.1a	*	33.7 ± 2.5	28.6 ± 0.1a	ns	0.8 ± 0.03	0.5 ± 0.0a	*	0.3 ± 0.01a	0.3 ± 0.08a	ns	20.7 ± 1.7c	19.4 ± 0.3c	ns
11591	125.4 ± 6.1a	111.2 ± 12.2b	ns	35.1 ± 3.4	38.8 ± 2.1b	ns	0.7 ± 0.09	0.9 ± 0.02b	ns	0.6 ± 0.1b	0.4 ± 0.03b	ns	11.1 ± 0.4a	10.3 ± 0.5a	ns
Avg.	128.4			35.2			0.8			0.5			13.8		
G × H	***			*			**			ns			***		

^[1]^ 3,5 DCQ: 3,5-Di-caffeoylquinic acid. In the columns of each harvest (H) time (winter and spring), means followed by the same letter are not significantly different at *p* ≤ 0.05. In the rows, least significant differences test indicates harvest time effect on each examined accession of *Parthenium argentatum*. G × H: interaction between genotype and season of harvest; ns, not significant; *** *p* ≤ 0.0001, ** *p* ≤ 0.001, * *p* ≤ 0.05.

**Table 5:** HPLC analysis of phenolic acids (mg g^−1^ DM) in twigs of the examined accessions of *Parthenium argentatum* harvested at 30 (winter) or 33 months (spring) after transplanting. (Means ± SD, *n* = 3, Fisher’s test).

	Neochlorogenic Acid			Chlorogenic Acid			Cryptochlorogenic Acid			Isovanillic Acid			Cynarin			3,5 DCQ ^[1]^		
Retention Time (min)	9.46			11.27			11.57			14.1			16.3			24.4		
	Winter	Spring		Winter	Spring		Winter	Spring		Winter	Spring		Winter	Spring		Winter	Spring	
AZ-1	0.6 ± 0.01b	0.4 ± 0.02b	*	29.7 ± 1.2c	34.0 ± 0.4c	*	0.3 ± 0.02	0.3 ± 0.01a	ns	0.3 ± 0.03a	0.2 ± 0.01a	*	1.5 ± 0.1a	0.9 ± 0.01	ns	18.8 ± 0.4b	20.8 ± 0.2ab	ns
AZ-2	1.04 ± 0.1c	0.4 ± 0.01b	*	33.0 ± 1.3d	22.8 ± 2.5b	*	0.3 ± 0.01	0.4 ± 0.05b	ns	0.7 ± 0.1b	0.3 ± 0.01b	ns	0.6 ± 0.1a	1.5 ± 0.3	ns	26.2 ± 0.8c	22.5 ± 1.8b	ns
P803	0.4 ± 0.02a	0.2 ± 0.02a	*	18.2 ± 1.5a	18.2 ± 1.7a	ns	tr	0.2 ± 0.0a		0.2 ± 0.02a	0.1 ± 0.0a	ns	1.4 ± 0.4a	1.9 ± 0.2	ns	15.2 ± 1.4a	15.3 ± 2a	ns
11591	0.5 ± 0.04b	0.3 ± 0.01a	*	25.8 ± 0.5b	18.3 ± 0.3a	*	tr	0.3 ± 0.02b		0.5 ± 0.1b	0.2 ± 0.08a	*	1.7 ± 0.8b	1.8 ± 0.2	ns	24.3 ± 2.4c	18.2 ± 0.5ab	*
Avg.	0.5			25.0			0.3			0.3*			1.4			20.2		
G × H	***			***			ns						ns			**		

^[1]^ 3,5 DCQ: 3,5-Di-caffeoylquinic acid. In the columns of each harvest (H) time (winter and spring), means followed by the same letter are not significantly different at *p* ≤ 0.05. In the rows, least significant differences test indicates harvest time effect on each examined accession of *Parthenium argentatum*. G × H: interaction between genotype and season of harvest. ns, not significant; *** *p* ≤ 0.0001, ** *p* ≤ 0.001, * *p* ≤ 0.05. Tr, trace quantities.

**Table 6:** Correlations (R^2^) established between antioxidant capacity (ABTS, DPPH), total phenolics (TotP), non-tannic phenolics (NTP), tannic phenolics (TP), total flavonoids (TotF) and phenolic acids in leaves and twigs of the examined accessions of *Parthenium argentatum* harvested at 30 (winter) and 33 months (spring) after transplanting.

	Leaves						Twigs					
Phenolic acids	ABTS	DPPH	TotP	NTP	TP	TotF	ABTS	DPPH	TotP	NTP	TP	TotF
Neoclorogenic	0.3097 **	0.4045 **	0.4208 **	0.4418 **	0.0079	0.1232	0.2926 *	0.1405	0.0857	0.3733 *	0.0627	0.0652
Chlorogenic	0.0236	0.0873	0.2381 *	0.1544	0.0378	0.5341 ***	0.4853 **	0.4853 **	0.4030 **	0.6734 ***	0.0027	0.5139 ***
Cryptochlorogenic	0.0429	0.0334	0.1829 *	0.1827 *	0.0031	0.1127	0.2365 *	0.2690 *	0.2504 *	0.0901	0.2017 *	0.3535 *
Isovanillic	0.1736 *	0.0009	0.0028	0.1388	0.1386	0.0173	0.2475 *	0.1212	0.0814	0.2373 *	0.0135	0.0335
Cynarin	-	-	-	-	-	-	0.2518 *	0.2511 *	0.1364	0.3158 *	0.0046	0.1828 *
3,5-DCQ	0.0607	0.0102	0.0004	0.1266	0.1932 *	0.0136	0.4843 **	0.3800 **	0.3251 *	0.4339 **	0.0192	0.2497 *

*** *p* ≤ 0.0001, ** *p* ≤ 0.001, * *p* ≤ 0.05.

**Table 7:** Phenolic compounds identified in *Parthenium* genus and members of the Asteraceae family screened in leaf and twig extracts of guayule by HPLC-DAD.

		Rt (min)	λ (nm)	References
1	Gallic acid	6.76	280	[23,45,46]
2	Pyrogallol	7.77	280	[45]
3	Chlorogenic acid	11.27	280; 330; 350	[12,24,33,46,47,48]
4	Catechin	11.47	280	[12,47,49]
5	Criptochlorogenic acid	11.57	280; 330; 350	[48]
6	Vanillic acid	14.18	280	[23,31,45,50]
7	Isovanillic acid	14.25	280	[12]
8	Caffeic acid	14.29	280; 330; 350	[23,31,45,48,49,50]
9	Syringic acid	14.85	280	[51,52]
10	Cynarin	16.4	280, 330,350	[12,47,53]
11	p-Coumaric acid	20.00	280; 330; 350	[23,48,50]
12	Rutin	20.27	280; 330; 350	[24,46,47]
13	Verbascoside	21.42	280; 330; 350	[26]
14	Luteolin 7-O-β-D-glucoside	21.61	280; 330; 350	[45,49]
15	Sinapic acid	21.88	280; 330; 350	[20]
16	Ferulic acid	21.98	280; 330; 350	[23,50]
17	1,5-Di-caffeoylquinic acid	24.19	280; 330; 350	[12,31,47,53]
18	3,5-Di-caffeoylquinic acid	24.37	280; 330; 350	[12,31,47,53]
19	Chicoric acid	24.18	280; 330; 350	[20]
20	Myricetin	27.40	280; 330; 350	[24]
21	3,4-DMCA	30.95	280; 330; 350	[20]
22	Luteolin	34.01	280; 330; 350	[33,45]
23	Quercetin	34.21	280; 330; 350	[33,46]
24	Cinnamic acid	35.52	280	[26]
25	Apigenin	37.31	280; 330; 350	[20,33]
26	Naringenin	37.41	280; 330; 350	[52]
27	Kaempferol	38.06	280; 330; 350	[11,24]
28	Mangostin	45.5	280; 330; 350	[54]
